# 3D printed optics with nanometer scale surface roughness

**DOI:** 10.1038/s41378-018-0015-4

**Published:** 2018-07-16

**Authors:** Nina Vaidya, Olav Solgaard

**Affiliations:** Electrical Engineering, Stanford University, 348 Via Pueblo Mall, Stanford, CA 94305-4090 USA

## Abstract

Complex optical devices including aspherical focusing mirrors, solar concentrator arrays, and immersion lenses were 3D printed using commercial technology and experimentally demonstrated by evaluating surface roughness and shape. The as-printed surfaces had surface roughness on the order of tens of microns. To improve this unacceptable surface quality for creating optics, a polymer smoothing technique was developed. Atomic force microscopy and optical profilometry showed that the smoothing technique reduced the surface roughness to a few nanometers, consistent with the requirements of high-quality optics, while tests of optical functionality demonstrated that the overall shapes were maintained so that near theoretically predicted operation was achieved. The optical surface smoothing technique is a promising approach towards using 3D printing as a flexible tool for prototyping and fabrication of miniaturized high-quality optics.

## 3D printing: smooth surfaces for optics

3D printing in conjunction with a polymer surface smoothing technique enables high-quality lenses with surface roughness of a few nanometers. 3D printing is an attractive process for on-demand optics, permitting fast and cheap fabrication of prototype optics with geometries that cannot be achieved via other approaches. However, the rough surface of most 3D printed optics introduces scattering sites, reducing optical performance. Nina Vaidya and Olav Solgaard from Stanford University now report that applying a UV curable polymer mixture to the surface of as-printed parts imparts surface roughness of a few nanometers, compared to tens of microns for as-printed parts. The fabrication of parabolic reflective lenses—mirrors, solar concentrator arrays and immersion lenses—demonstrates that 3D printing can be used for optics with high surface quality.

## Introduction

Additive manufacturing and three-dimensional (3D) printing have improved access to and flexibility of high-quality fabrication technology with profound impact on a number of industries^1^, including automotive, electronics^2–4^, aerospace, bio-engineering^5,6^, and microfluidics^7^. Complex to fabricate optical devices^8–10^ and systems can similarly benefit from the ability of 3D printing to create low-cost structures of nearly arbitrary shape^11,12^. Optical designs are often complicated by the need to conform to shape constraints imposed by fabrication technologies, e.g., spherically polished lenses, flat imagers, and shapes that can be efficiently molded and cast. Other cost driving constraints concern integration of different devices made in different materials and by different processes. By lifting these constraints and providing integrated solutions that can be manufactured cost effectively even in small series, 3D printing has the potential to revolutionize design, prototyping, and fabrication of optical systems. To realize this potential, 3D printing must be refined to meet the needs of optical devices^13–15^.

3D printing is implemented in a variety of technologies including Direct Metal Laser Sintering, extrusion as in Fused Deposition Modeling and Fused Filament Fabrication, lamination as in Laminated Object Manufacturing, Drop-on-Demand (DoD) inkjet type printing where a wax like substance is jetted as micro-droplets, and polymerization through StereoLithography Apparatus (SLA). In each of these technologies, 3D structures are printed on a flat substrate by curing lines of material or rows of drops of material and building up the structure layer by layer. For optics, this fabrication approach leads to three challenges: (1) the printed material has density variations that results in excess scattering, (2) surfaces that are not parallel to the initial substrate have steps corresponding to the layer thickness, and (3) the completed shape of the structure might deviate from the design.

In this paper, we demonstrate a 3D printing approach that directly addresses these three issues. We solve the scattering problem by focusing on reflective optics and on printed molds, and we solve the surface roughness and shape problems by developing a surface smoothing technology that removes surface roughness without changing the overall shape. Using SLA polymer and DoD wax 3D printers, we realized three different types of lenses that are complicated to manufacture in traditional ways and typically not found as off-the-self components: parabolic reflective lenses, i.e. mirrors, concentrator arrays, and immersion lenses. The complexity and plurality of parameters that characterize these types of optical devices make it impractical to offer them as standardized products. This necessitates custom designs and specialized fabrication for all but a few very large-volume applications. 3D printing therefore has the potential to become an invaluable fabrication method for such applications.

## Printing and surface smoothing

Optical parts were designed and simulated with ray tracing software. The shapes were reproduced in the STL (STereoLithography) file format, which is one of the standard file formats of 3D printing. The design files were printed both by our in-house printers and by commercial companies specializing in 3D printing services, so that several different printing technologies could be explored and compared. It was found that Stereolithography (SLA) printers and wax printers created the best optics compared to extrusion technologies. SLA printers create 3D structures by ultraviolet (UV) curing in a bath of polymer (poly-carbonate (PC) like), resulting in a layer resolution of about 50 μm from the printers we used. Wax printers, conventionally used to make jewelry molds, use DoD inkjet-like printing technology and the printer we used typically achieves layer resolution of 6.3 μm.

Surface shape errors are typically specified as an error in fractions of wavelength found through optical interference between a known nominal optical shape and the one manufactured. Apart from the shape, the higher frequency error, i.e. the nanometer scale surface roughness, is usually stated as a root mean square (rms) value. Assuming Gaussian distribution and subwavelength surface roughness, the fraction of light specularly reflected is given by^16^

1 $$\exp \left[ { - \left( {\frac{{4\pi {R}_q\cos \theta }}{\lambda }} \right)^2} \right],$$

where *R*_*q*_ is the rms roughness, *θ* is the incidence angle, and *λ* is the wavelength. Using this equation, we find that 3 nm surface roughness results in a scattering loss of less than 1.5% at normal incidence for wavelengths above 300 nm. Typically, a precision-quality diamond turned metallic mirror has a surface roughness of 5 nm^17^.

We tried a number of smoothing techniques, including flame polishing, acetone vapor polishing, spraying of polymer coatings, and mechanical polishing. None of these methods create the nanometer scale smooth surfaces required for optical applications. To meet this surface roughness criterion, we coated the printed optics with a UV curable polymer mixture consisting of methacrylates, acrylates, and urethane based polymers^18^. This gel resulted in smooth and tough films that adhered well to the printed surfaces. When compared to a heat cure, a UV cure minimizes shrinkage of the polymer, which maximizes surface smoothness and conformal coverage. The detailed process is as follows:

1. Rinse the 3D printed part with water and detergent. Wash with DI (de-ionized) water and blow dry. Leave to completely dry in low temperature oven.
2. Place part in vacuum to degas for a few hours.
3. Coat a thin layer of gel (UV curable polymer mixture) on the surface of the 3D printed part with a fine brush.
4. Place in vacuum chamber to get rid of any air trapped in the printed material, in the gel layer, or in between the printed surface and the gel so that the gel can fill in any pores or depressions to make smooth surfaces.
5. If needed for conformal coverage, use gravity or spinning to remove excess gel. Let gel flow under gravity by placing the optics flat on a stand. Spin at around 1400 rpm for 3–5 min while the gel is still un-cured. Brush off excess gel at the edge of the frame/support.
6. UV cure the finished gel surface for a couple of minutes, with the exact time depending on the size of the part.

## 3D printed mirrors

Surface smoothing for reflective lenses, i.e. mirrors, is complicated by the requirement that the smoothness of the surface must be preserved in vacuum so that a mirror layer (metal or di-electric) can be deposited. This excludes a number of popular 3D printing materials, because they outgas too much in vacuum to allow metallization. We found that neither SLAs nor wax printers suffer from this problem.

As a base line test, flat blanks were printed in an SLA printer, smoothed, and then metalized and tested. The as-printed flat surfaces consisted of continuous printed layers (no steps) and were therefore relatively smooth with a measured surface roughness of 70 nm rms. Our smoothing technique reduced this by more than one order of magnitude to 2.3 nm rms, measured using atomic force microscopy (AFM). After smoothing, a seed layer of 150 Å Ti (titanium) followed by 1000 Å Al (aluminum) were evaporated onto the printed surfaces. The resulting 3D printed mirrors behaved as expected for flat Al across the 200–1800 nm wavelength range with no detectable anomalies due to surface roughness, verified by spectrophotometer specular reflection measurements at several locations and different incidence angles. The difference between this UV gel smoothing method and other optical coatings is that it is designed for rough surfaces, improving the rms surface roughness from microns to subwavelength scale of less than 3 nm, unlike for example, a thin anti-reflection coating that needs a flat smooth substrate like polished glass.

**Fig. 1:**
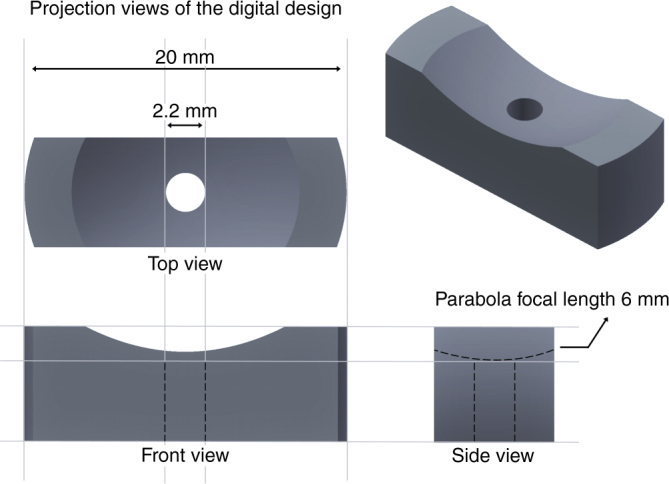
**Parabolic mirror’s stereolithography (.stl) file format of target design**

To verify that our smoothing process works on curved surfaces without significantly changing their shape, we fabricated a complex parabolic mirror, as shown in Fig. 1, that is designed to be the focusing element of a dual axes confocal microscope^19^. The mirror has a focal length of 6 mm, a 10 mm outer radius, and a center hole of 1.1 mm radius. Several parabolic mirrors were made both with the SLA and wax printers, and smoothed by our gel smoothing technique.

**Fig. 2:**
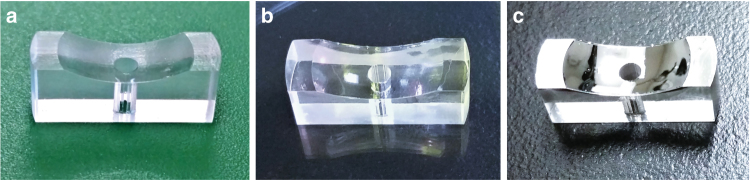
3D printed parabolic mirrors at different stages of the fabrication process. As printed (**a**), after smoothing (**b**), and the completed mirror after smoothing and Al deposition (**c**)

**Fig. 3:**
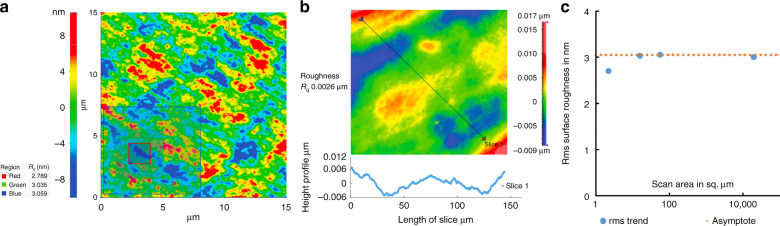
Surface roughness characterization of the 3D printed and smoothed mirrors. AFM data (**a**) and profilometer measurement (**b**) of surface roughness on the 3D printed parabolic mirror. The rms surface roughness asymptotically approaches a limiting value of 3 nm as the measurement area increases (**c**)

The curved surfaces led to much higher surface roughness for the as-printed parabolic mirrors than for the flat mirrors. Measurements showed that the as-printed surface roughness was on the order of a couple of micron rms. This improved by three orders of magnitude to 3 nm rms after smoothing and metallization with a 150 Å Ti base seed layer followed by 1150 Å Al layer. Figure 2 shows the 3D printed parabolic mirror at three different stages of completion: (a) as-printed, (b) after smoothing, and (c) after metallization. The improved surface quality was verified by AFM over small areas (Fig. 3a) and by profilometer measurements over larger areas (Fig. 3b). As expected for surfaces with bounded outliers, we found that the measured surface roughness asymptotically converged to a finite rms value as we increased the measured area (Fig. 3c)^20^.

**Fig. 4:**
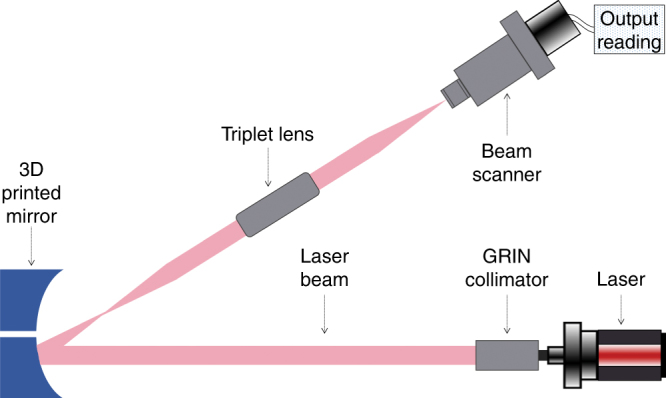
**Beam path in the optical test setup to measure the mirror’s beam size at the focus**

The parabolic mirrors were characterized in the focusing setup shown in Fig. 4. The size of the focused beam was measured using a beam scan with a rotating slit to record the focusing performance of the mirrors. A red laser of wavelength of 675 nm (*λ*) with a GRIN collimator was used to illuminate the mirror with an incident beam area of diameter 0.55 mm. The theoretical focal length of this parabolic mirror is 6 mm (*f*), so its focus was replicated at the beam scan using a telescope triplet lens. Equation for Gaussian beam focusing of a collimated beam (flat wavefront on the lens)^21^ gives the diffraction-limited beam radius at the focus of the parabolic mirror:

2 $$\omega _{\mathrm {focus}} = \frac{{f \times \lambda }}{{\pi \times \omega _{{\mathrm {lens}}}}} = \frac{{6\,{\mathrm {mm}} \times 0.675\,{\upmu \mathrm {m}}}}{{\pi \times \frac{{0.55\, {\mathrm {mm}}}}{2}}} \approx 4.69\, {\upmu} {\mathrm{m}}$$

The diffraction-limited beam diameter is then 9.4 μm.

**Fig. 5:**
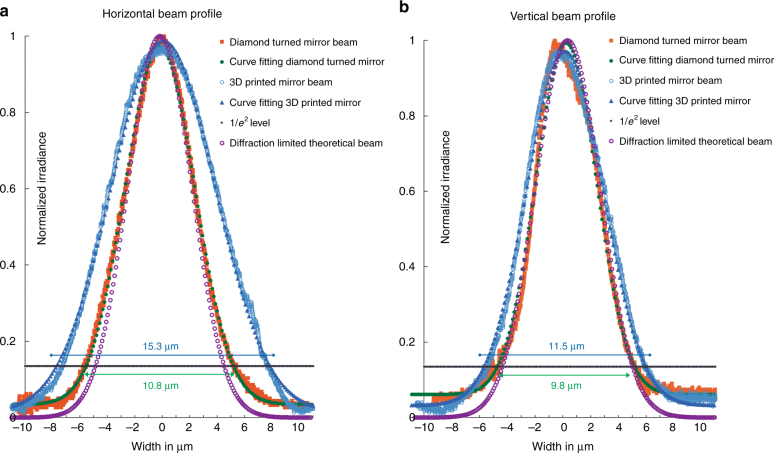
Comparison of beam focus reflected from 3D printed parabolic mirror and beam focus from an identical diamond turned parabolic mirror. Gaussian curve fitting was done on the experimental data and the beam waist shown at the 1/*e*^2^ level. The horizontal beam profiles are shown in (**a**) and the vertical ones in (**b**)

A metallized as-printed 3D parabolic mirror without the intermediate smoothing step created a matte and non-specular surface which was not of optical quality as there was unacceptable light scatter from the micron scale surface roughness; most of the incident light was scattered as predicted by Eq. (1). Hence, to quantify the imaging properties of the printed and smoothed parabolic mirrors, beam profiles measured at their focus were compared to beams focused by an identical Al metal diamond turned parabolic mirror. The horizontal and vertical beam sizes of the 3D printed mirror and the diamond turned mirror are shown in Fig. 5. In each case, the raw experimental data are plotted along with Gaussian curve fitting of the data to find the 1/*e*^2^ beam waist. In Fig. 5a which displays the horizontal focal plane, the 3D printed mirror created a spot size with beam waist of 15.3 μm as seen in the curve with green circles, compared to 10.8 μm for the diamond turned mirror in the curve with blue triangles. In Fig. 5b which represents the vertical plane, the 3D printed mirror created a spot size of 11.5 μm as seen in the curve with green circles, compared to 9.8 μm for the diamond turned mirror seen in the curve with blue triangles. The diffraction-limited theoretical Gaussian beam curve at the focus, having a beam diameter of 9.4 μm given by Eq. (2), is represented as the curve with purple circles in Fig. 5a, b for comparison with experimental results. The vertical beam sizes are closer to the theoretical value of 9.4 μm.

The larger difference in the horizontal and vertical beam widths for the 3D printed mirror than for the diamond turned mirror measurements shows that there is added astigmatism due to the 3D printed mirror shape apart from astigmatism in the test setup, which is common to both measurements. This extra astigmatism was most likely due to the way the 3D printer lays down the features or the smoothing process as the astigmatism was much lower while testing the diamond turned mirror in the same experimental setup. The parabolic profile parallel to the 3D printed path/sequence followed the desired analytical shape in the digital SOLIDWORKS file more accurately. The profile shape perpendicular to the 3D printing sequence fell short of having the desired ideal curve due to the additive nature of 3D printing as the parabolic shape in the perpendicular direction is constructed in stepped lines of cured resin/row of micro-droplets. The astigmatism may have also been introduced at the process step after the 3D printing, while smoothing and curing the gel. Even with these fabrication non-idealities, Fig. 5 demonstrates that a close to theoretically predicted diffraction-limited spot in the range of microns was created with mm-scale aperture size.

## Solar concentrator arrays

To demonstrate the ability of 3D printers to create unusual shapes a solar concentrator array was designed and fabricated. The concentrators have a graded-refractive index profile from a low-index input aperture to a high-index smaller output aperture that allows passive, loss-free concentration by a factor of RI^2^, where RI is the refractive index on the output, provided that the output is in optical contact with the optical absorber^22^. In operation, light rays enter the concentrator in a low-index medium, curve towards the normal, reflect from the sidewalls, and concentrate on the smaller output aperture in a high-index medium.

**Fig. 6:**
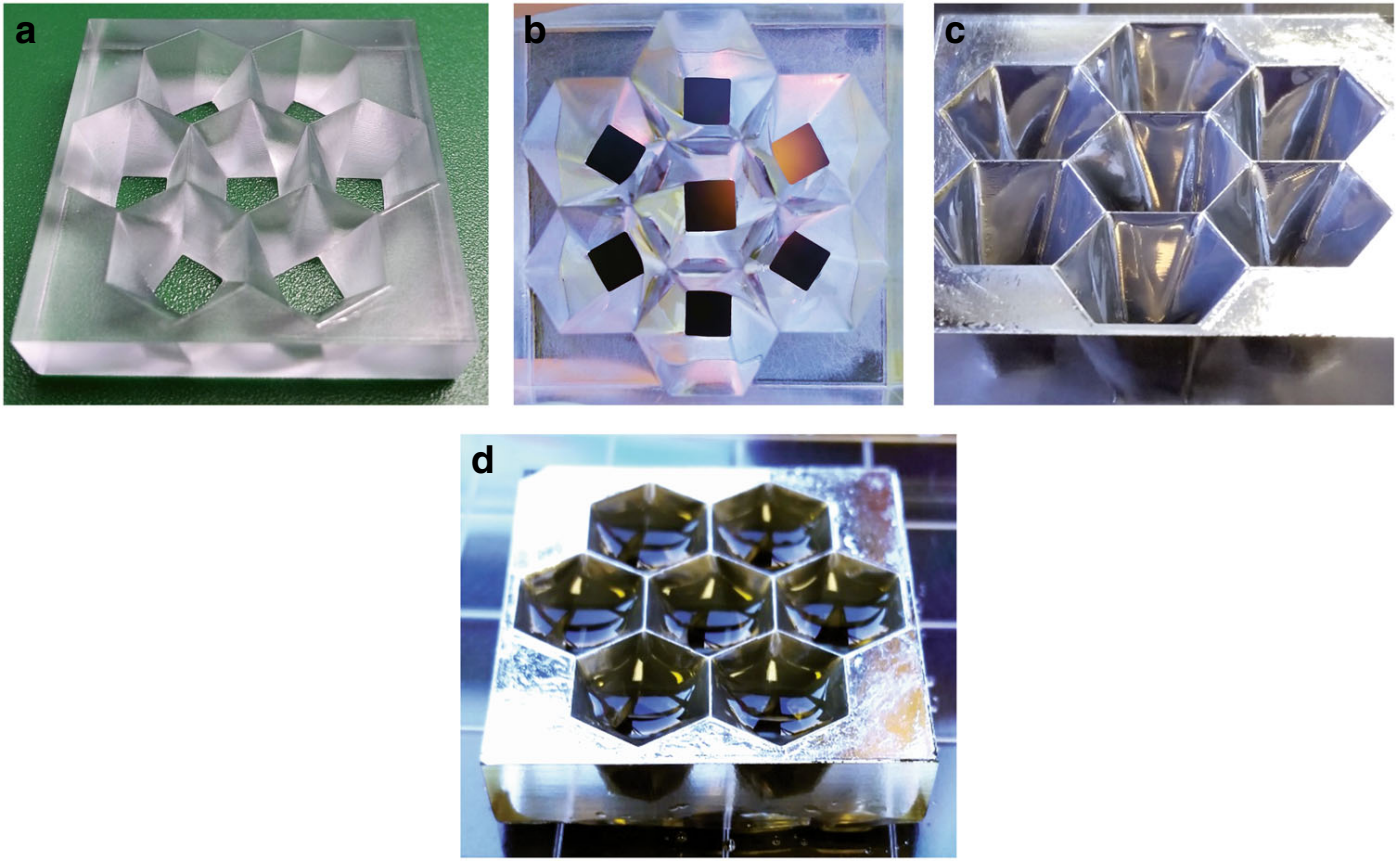
Tiled solar concentrator lens array made by 3D printing followed by smoothing. The input side is arranged as a tileable array of hexagons that along the length of the concentrators gradually morph into squares on the output side. The smaller squares at the output allow smaller solar cells to be used to convert the concentrated power. The molds were filled with graded-index polymers to complete the concentrator array. Figures **a**–**d** show the process flow from as-printed part to the completed concentrator array

Our prior demonstrations of these graded-index passive solar concentrators were based on fabrication of polymer and glass concentrators^23,24^ that required elaborate machining. 3D printing simplifies the fabrication and enables tileable input surfaces and flexible array design (Fig. 6). We designed a hexagonal tileable input surface with smaller square outputs for bonding with solar cells. The input hexagons had sides of 6.2 mm and transitioned to square outputs of side 4.5 mm over a height of 8 mm, creating a geometrical concentration of 5 suns. The 3D printed sidewalls had a roughness of the order of microns and the UV curable gel (same as the smoothing process described earlier in the paper) was used to make it optically smooth with a measured rms roughness of 2 nm.

Figure 6 shows the fabrication process. The striations on the 3D printed sidewalls that are seen in Fig. 6a were removed by the gel smoothing process resulting in smooth surfaces as seen in Fig. 6b. The smoothness of the surfaces was preserved after the metallization process (150 Å Ti seed layer followed by 1150 Å Al) as seen in Fig. 6c. To reduce clusters, microcracks, and haziness, the Al deposition was done as close as possible to normal to the surface, especially for sidewall features/corners. The solar concentrator array was completed by filling it with optical-grade polymer layers as seen in Fig. 6d. UV curable transparent optical polymers from Norland Products, Inc. of refractive index 1.56, 1.54, 1.53, 1.52, 1.50, and 1.46 in that order were used as the graded-index layer profile to fill the reflective mold. Due to mixing at the boundary between the cured and the newly deposited layer, the actual index profile had smooth transitions between discrete indices.

**Fig. 7:**
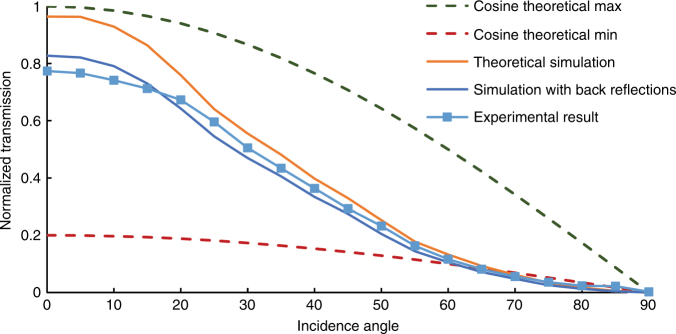
**Experimental performance of the 5 Sun (area ratio) concentrator array across different incidence angles in agreement with the simulations**

Optical transmission of the concentrator array was measured under a solar spectrum generator at different incidence angles. A non-corrosive index matching layer of refractive index 1.6 and a thickness of 0.5 μm was used for optical contact between the last layer of the array and the solar detector. Our ray tracing simulation model includes the six graded-index layers, the index matching layer, and the back reflections at the detector surface. Comparison between simulations and experimental results in Fig. 7 shows that the array demonstrated a passive concentration of 5 suns and followed the cosine theta theoretical maximum across an acceptance angle of about 40°.

## 3D molding and casting

**Fig. 8:**
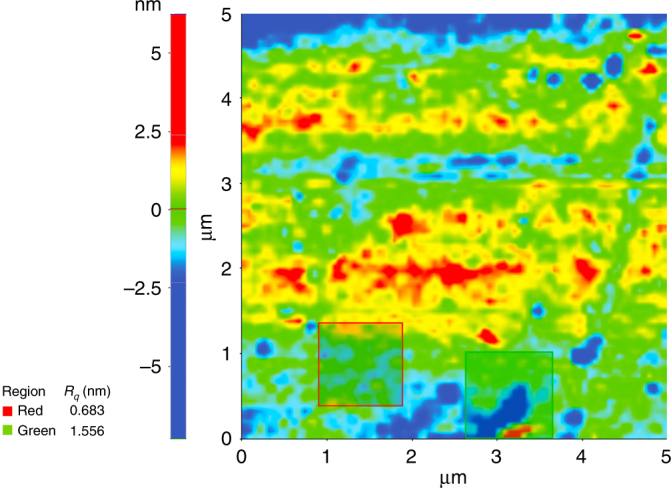
AFM of index matched immersion lens made with PDMS, released from 3D printed smoothed mold. Rms surface roughness (*R*_q_) is on average 1.4 nm

To create transmission lenses without excess scattering through density variations, we made 3D printed molds for immersion lenses used in microscopy of biological samples. Such lenses should have low index to match the index of most biological tissue, and should ideally be disposable so cleaning is not necessary. We used 3D printed molds and gel smoothing to fabricate polydimethylsiloxane (PDMS) hemispherical immersion lenses. Molds were 3D printed as hemisphere cups and the mold surfaces were smoothed using the UV gel smoothing technique. At this stage, the mold was filled by PDMS monomers and heat cured (45 °C for 24 h). Once the PDMS was completely cured, the PDMS parts released from the 3D printed mold’s smoothed surface by applying a small amount of pressure at the edges using a flat tip tweezer so as not to damage the lenses. The mold’s smooth surface was compatible with PDMS to be heat cured in contact with the mold’s previously UV cured gel surface and peeled off with ease. The surface roughness of the 3D printed and smoothed lens molds and of the released PDMS lens surfaces were measured and compared. AFM measurements verified that the surface roughness of the PDMS parts was 1.4 nm, i.e. essentially the same as the mold surface roughness (Fig. 8).

To verify the shape of the PDMS hemispheres, their focal lengths were experimentally measured with a laser beam of wavelength of 632.8 nm expanded over the lens apertures and a beam profiler on a translational stage. Several different sizes of hemispherical lenses were fabricated and tested, including lenses of 3, 4.75, and 7 mm radii, having measured focal lengths of 7.0, 11.24, and 16.57 mm, respectively. The nominal focal lengths calculated for these radii are 7.10, 11.33, and 16.62 mm using the PDMS Sylgard 184 refractive index at the test wavelength 632.8 nm of 1.4225. In each case, the experimentally measured focal lengths deviated by less than 1.5% from the nominal focal lengths, showing that the lenses have the desired hemispherical shapes.

## Discussion and conclusions

Our results show that the inherent surface roughness of 3D printed structures can be reduced to meet the criteria of high-quality optics by a UV gel smoothing technique that creates nanometer smooth surfaces. Measurements showed a dramatic reduction in surface roughness from tens of microns of the as-printed surfaces to less than 3 nm after the smoothing process. As we increased the measured area of the smoothed surfaces of the 3D printed parts, the rms roughness reached an asymptote of 3 nm, verifying the effectiveness and scale of the smoothing technique. In addition, these smooth surfaces have very low out gassing so they can withstand vacuum and different thin films can be deposited, which preserve the surface roughness of the smoothed gel surfaces. The 3D printed structures and their deviation from the designs were experimentally evaluated and it was found that the completed shapes approximated the designs with good fidelity.

Our experiments show that SLA and wax printers created better optical devices than the other printing technologies that were tested. The melting temperature of the printed wax is comparable to the polymers used in the SLA printer, but the wax is brittle at room temperature, so an added benefit of the gel smoothing technique is that it protects surfaces and yields tough and robust parts.

To demonstrate the versatility of the technique several different types of optics were fabricated, including parabolic mirrors, solar concentrator arrays, and immersion lenses. Imaging with 3D printed parabolic mirrors were comparable to a diamond turned metal mirror and nearly diffraction-limited spot sizes were measured with modest incidence apertures. Solar concentrator hexagonal arrays were made using 3D printing and they demonstrated 5 suns concentration across an acceptance angle of 40°. PDMS immersion lenses were made with nanometer smooth surfaces released from 3D printed molds.

The variety of fabricated devices shows that the described technology produces optics that are easy to fabricate, low cost, customizable, lightweight, and low on material waste due to the additive nature of 3D printing. This demonstrates that 3D printing, along with UV curable polymer surface smoothing, has the potential to become an important fabrication tool for high-quality optics and enable custom optical systems that are simpler, lighter, and lower cost than systems made using traditional technologies.
